# Functional magnetic resonance imaging of depression: a bibliometrics and meta-analysis

**DOI:** 10.1186/s12991-024-00525-x

**Published:** 2024-10-24

**Authors:** Xiaotong Wang, Xi Nie, Feng Zhang, Yuhan Wei, Weiting Zeng, Yuchuan Zhang, Haixiong Lin

**Affiliations:** 1 School of Acupuncture-Moxibustion and Tuina, Beijing University of Chinese Medicine, Beijing, 100029 People’s Republic of China; 2 Center for Neuromusculoskeletal Restorative Medicine & Institute for Tissue Engineering and Regenerative Medicine, The Chinese University of Hong Kong, 999077 Hong Kong SAR, People’s Republic of China; 3 Department of Orthopedics, Ningxia Hui Autonomous Region Chinese Medicine Hospital and Research Institute of Chinese Medicine, Ningxia Medical University, Yinchuan, 750021 People’s Republic of China

**Keywords:** CiteSpace, Hotspots, Brain regions, Pathogenesis

## Abstract

**Objectives:**

This study aims to reveal the current knowledge map, research hotspots of functional magnetic resonance imaging (fMRI) studies on depression, as well as identify the brain regions associated with depression.

**Methods:**

CiteSpace was conducted to analyze the publication outputs, country, institution, cited journals, author and cited author, references, keyword cocurrence and burst keywords of fMRI studies in depression from 2010 to 2024. And a meta-analysis of fMRI was used to identify brain regions associated with depression using Neurosynth.

**Results:**

A total of 4,049 publications were included, and Gong Qiyong was the most prolific authors. Neuroimage, Biological Psychiatry, and Human Brain Mapping were prominent journals. Default mode network (DMN), prefrontal cortex, amygdala, and anterior cingulate cortex were the popular keywords. The fMRI studies on depression have mainly focused on major depression, especially the DMN. Functional connectivity and regional homogeneity of brain regions were research hotspots. The meta-analysis revealed significant differences in brain regions between patients with depression and healthy controls, including the Amygdala_L, Insula_R, Frontal_Inf_Oper_R, Cingulum_Post_L, Putamen_L, Thalamus_R, Angular_L, Precuneus_R, Frontal_Sup_R, Occipital_Inf_L.

**Conclusions:**

This study sheds light on key issues and future directions in fMRI research on depression, elucidating the brain regions related to depression.

**Graphical Abstract:**

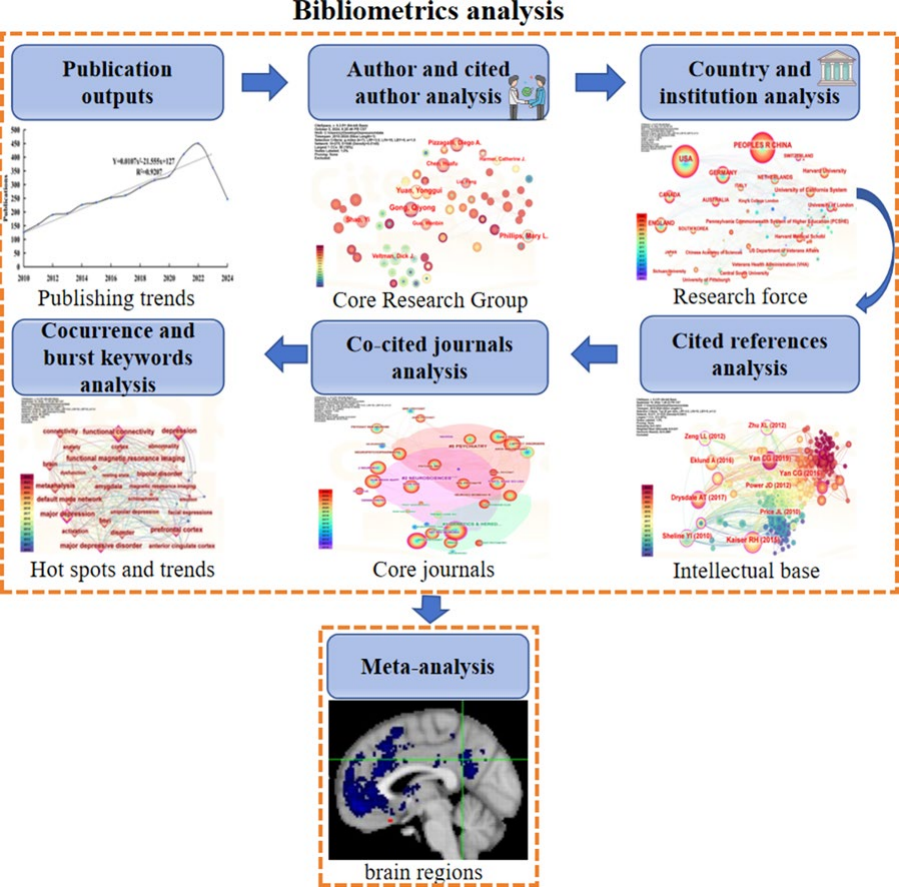

**Supplementary Information:**

The online version contains supplementary material available at 10.1186/s12991-024-00525-x.

## Introduction

Depression is a complex mental illness characterized by a persistent low mood, sluggish thinking, reduced mental activity, feelings of inferiority, negativity, and even suicidal tendencies and behaviors [1]. Depression has destructive effects on health and daily functioning [2]. In addition, depression is also characterized by a high rate of recurrence, disability, and mortality [3]. It is reported that the global incidence rate of depression is 4.4% [4]. According to the trend of depression incidence, depression will become one of the top three disease burdens globally by 2030, especially in low- and middle-income countries [5]. Current methods of screening and assessing depression rely almost exclusively on clinical interviews and self-report scales [6]. And the clinical diagnosis of depression and the currently used treatment methods both lack objective biological markers [7]. With the development of neuroimaging techniques, more and more researchers are using fMRI to study the structural and functional characteristics of the brains of patients with depression, in the hope of identifying neuroimaging biomarkers for depression [8].

The fMRI technique has the advantages of high spatial resolution, no radiation, and non-invasiveness, which enables researchers to observe changes in brain activity in patients with depression non-invasively [9]. By comparing the brain activation patterns of patients with depression and non-patients, scientists have found that depression is associated with functional abnormalities in brain regions such as the prefrontal cortex, amygdala, and cingulate gyrus [10]. In addition to studying the activation patterns of specific brain regions, recent studies have also focused on the connectivity patterns between brain networks. Previous studies have shown that there are differences in the connectivity patterns and functional networks within the brains of patients with depression, particularly in networks involved in emotion regulation, cognitive control, and reward systems [11].

Bibliometrics is a quantitative research method based on publication, citation, and text data, which is a useful method for describing the trends in the development of science fields [12]. The results of bibliometric analysis not only include descriptive statistics, but also network analysis of keywords, citations, authors, and institutions and their relationships. It examines the frequency, correlation, centrality, and clustering of author and text data, and is often used by scholars to explore the evolution patterns of a topic, publishing trends, author citation networks, and other key information [13]. In order to understand the development trend of fMRI in depression and provide reference for finding neuroimaging biomarkers for depression, a bibliometric mapping analysis was conducted to explore the knowledge structure of this field.

The idea of meta analysis in neuroimaging is very similar to that of meta analysis in clinical therapy, but the goals of application are different. Neuroimaging studies report the peak coordinates of activated brain regions (i.e., activation peak points, foci) under several contrast conditions [14]. From this perspective, looking for consistency in activation locations across multiple studies is a feature of current neuroimaging research. Therefore, meta-analysis based on coordinate data has gradually become the mainstream of meta-analysis of neuroimaging data. Therefore, a meta-analysis was further performed on current fMRI results comparing patients with depression to healthy controls to identify differences in brain regions between the two groups.

## Methods

### Data retrieval and acquisition

Literatures were retrieved online through SCI-E of the WoS Core Collection. The following search terms and synonyms were used in data retrieval strategy with a time span ranging from January 1, 2010 to September 15, 2024: (TS = (depression) AND TS = (Functional magnetic resonance imaging). Language was restricted to English. However, no document type, or data category was restricted. All retrieved studies were downloaded in full text in the same day to avoid changes in citation counts caused by database updates.

### Analysis tool

CiteSpace V6.3.1 was used for bibliometric analysis. A visual knowledge maps consists of nodes and links. The colors of nodes and lines suggest different years. The different nodes in a map indicate different elements such as countries, institutions, journals, authors, cited authors, cited references, and keywords, while links between nodes suggest collaboration, cooccurrence or cocitation relationships. The citation year ring represents the citation history of this article. The color of the citation ring represents the corresponding citation time. The thickness of an annual ring is proportional to the number of citations in a time zone [15]. The centrality of the node is a graph theory that quantifies the importance of points in the network [15]. Nodes with high centrality are often considered to be important turning points or key points in the field [16].

We conducted a meta-analysis of fMRI studies on patients with depression using Neurosynth (http://www.neurosynth.org). Neurosynth is a platform for large-scale, automated synthesis of fMRI data including 507,891 activations from 14,371 studies. As an automated brain-mapping framework Neurosynth applies text-mining and meta-analysis techniques to generate a large database of mappings between neural and cognitive states [17]. This database has the capability to automatically extract coordinates of brain regions relevant to the diseases of interest, allowing for a comprehensive automated meta-analysis.

### Analysis methods

Data was imported into the online “WoS Core Collection Literature Analysis Report” (https://bibliometric.com/) to obtained the number of annual publications and document types. Journal Impact factor, Journal Citation Indicator and quartile in category of journals were derived from the Journal Citation Report (JCR) 2023 standard.

Data was imported into the CiteSpace software to identify collaborations, hotspots, frontiers and trends in functional magnetic resonance imaging of depression by analyzing collaborative networks between countries-institutions/authors, exploring associations between journals/cocited references, and capturing keywords with strong citation bursts. The parameters of CiteSpace were set as follows: time slicing (2000–2024), years per slice (1), term source [title, abstract, author keywords, keywords plus (ID)], pruning (pathfinder and pruning sliced networks), and visualization (cluster view-static and show merged network). Node types were selected based on the purpose of analysis, including author, institution, country, keyword, reference, cited author and cited journal. The brain region coordinates were extracted from the literature, and a meta-analysis was conducted to determine the different brain regions between depressed patients and healthy controls.

The Neurosynth database searched for brain regions associated with depression by using "depression" as a search term. The automated parser extracts activation coordinates from published neuroimaging articles. The full text of each article was parsed, and each was tagged with a set of terms that frequently appear within it. In addition to generating statistical inference maps (i.e., z-value and p-value maps), posterior probability maps were also calculated. These maps indicate the likelihood of a given term being used in the study if activation is observed at a specific voxel. To produce the uniformity test map, we applied a false-discovery rate adjusted p-value of 0.01. Subsequently, the xjView toolbox V.8 in MATLAB (https://www.alivelearn.net/xjView/) was used to select the top ten brain regions associated with depression based on voxel ranking [18].

## Results

### Analysis of publication outputs

**Fig. 1:**
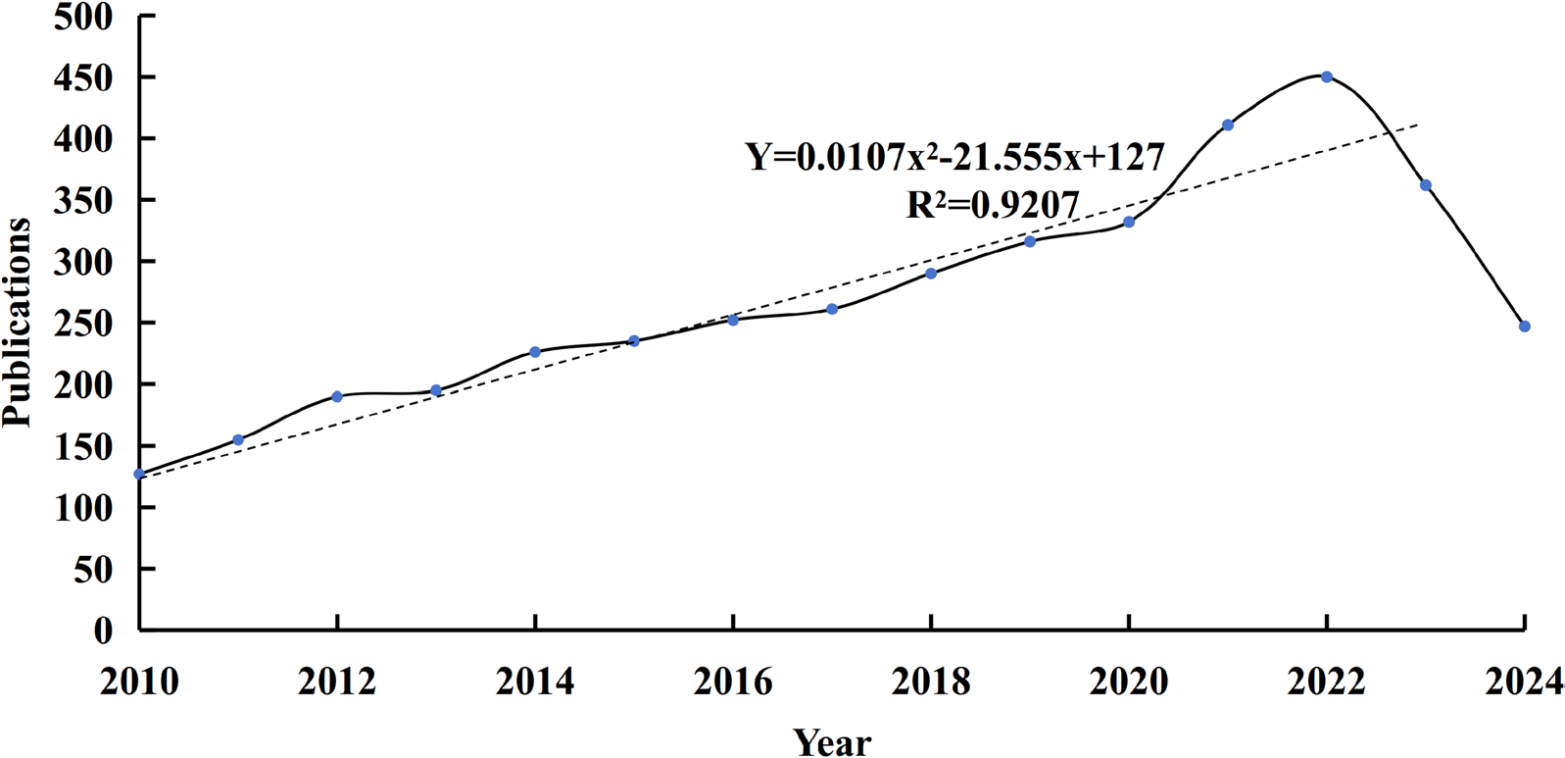
The number of functional magnetic resonance imaging of depression publications indexed by SCI-E from 2010 to 2024. The solid line is the number of publications per year, and the dotted line is the trend line of the simulated number of publications per year

From 2010 to September 2024, a total of 4,049 articles on fMRI studies of depression were published in WoS. The number of publications from 2010 to 2024 were:127, 155, 190, 195, 226, 235, 252, 261, 290, 316, 332, 411, 450, 362, 247. The model for fitting the annual growth data from 2010 to 2023 is y = 0.0107x^2^−21.555x + 127 (R^2^ = 0.9207, X = Year) (Fig. 1). The number of publications has remained stable at over 300 since 2018, with the highest number of publications in 2022. Therefore, scholars around the world were paying increasing attention to depression.

### Analysis of author and cited author

**Fig. 2:**
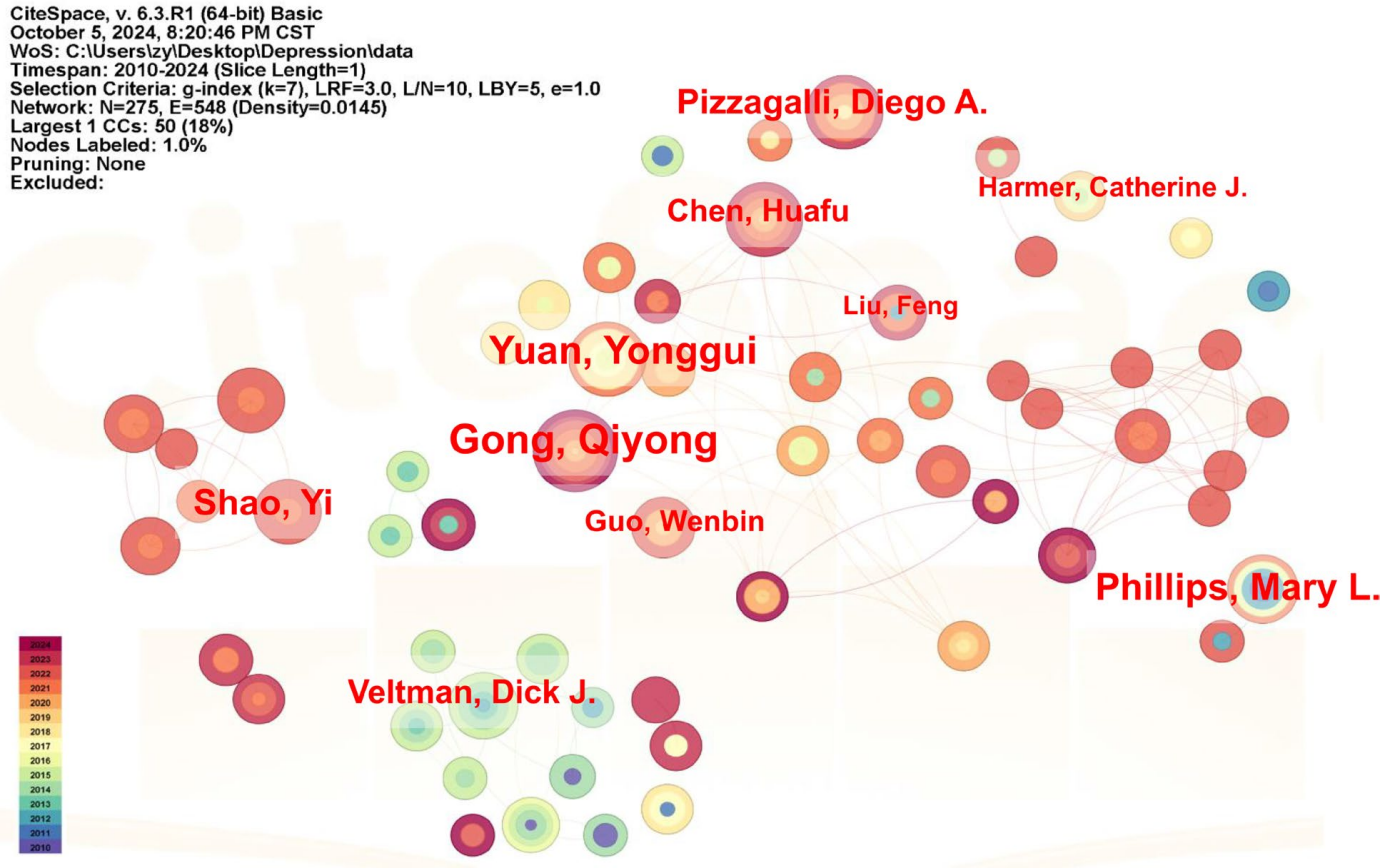
A coauthor map related to functional magnetic resonance imaging of depression research from 2010 to 2024. The color represents the year, the size of the circle represents the number of publications, and the line represents the collaboration between the authors

Core authors are a vital force in driving academic innovation and disciplinary development, as well as an important factor in enhancing the academic impact and competitiveness of journals. Price's Law is an important metric for bibliometric evaluation that identifies potential core author group, and he proposes that half of the studies on a topic will be contributed by the square root of the total number of authors [19]. According to Price's Law, half of the studies in the field of fMRI for depression were contributed by 22 high-productive authors. However, these 22 authors contributed only 704 articles, accounting for 17.39% of the total number of papers in this field, which is lower than the 50% threshold set by Price's Law. This indicates that the core author group in the field of fMRI research on depression has not yet formed. Among the high-productivity authors, Gong Qiyong has the highest number of publications, with a total of 55 papers published from 2010 to September 2024, receiving 2,649 citations and an average of 48.16 citations per paper (Supplementary Table 1). Gong Qiyong discovered the coupling between brain structure and function, proposed the "brain-behavior dyad" hypothesis, and used this theory to explain the mechanism of severe mental illnesses, such as discovering that the brain connectivity network of first-episode severe depressive patients who have not received drug treatment is disrupted using graph theory analysis [20]. Yuan Yonggui ranks second with 45 publications and 865 citations, with an average of approximately 19.22 citations per paper. Gong Qiyong and Yuan Yonggui have also collaborated on a research article titled "Altered resting-state dynamic functional brain networks in major depressive disorder: findings from the REST-meta-MDD consortium." (Fig. 2). Mary L. Phillips, whose research paper had the highest average citations per publication, used fMRI to investigate the blood oxygen level-dependent response to facial expressions of fear and disgust in patients with depression [21–23]. Immediately followed by Harmer Catherine J and Gong Qiyong. Harmer Catherine J mainly studied the effects of antidepressants (Selective Serotonin Reuptake Inhibitors, SSRIs) on the neural responses in the medial orbitofrontal cortex, amygdala, and prefrontal cortex of depressed patients [24–26], in particular, SSRIs treatment was shown for the first time to reduce the neural processing of both rewarding and aversive stimuli with the highest number of citations of 246 citations [24–26].

### Analysis of co-cited journals

Clustering the cited journals resulted in three fields: neurosciences, psychiatry, genetics & heredity (Supplementary Fig. 1). The neurosciences journals, such as Human Brain Mapping and Journal of Neuroscience, focus on neuroimaging and neuroscience research. Journals in the pink cluster mainly belong to the field of psychiatry, such as American Journal of Psychiatry, and Journal of Affective Disorders, which cover all disciplines and research areas related to the pathophysiology and treatment of mental illness. Journals in the green clusters mainly belong to the fields of genetics & heredity, which focus on the basic and clinical contributions of specific disciplines and research areas.

The Table 1 displays the top ten journals by cocitation count: Neuroimage, Biological Psychiatry, Human Brain Mapping, American Journal of Psychiatry, Journal of Affective Disorders, Proceedings of the National Academy of Sciences of the United States of America, Journal of Neuroscience, Plos One, Neuropsychopharmacology, Cerebal Cortex. The journal with the highest number of cocitations was "Neuroimage," which published content covering, but not limited to, the following areas: analysis methods, functional MRI acquisition and physics, computational modeling and analysis, anatomy and physiology, cognition and aging, social neuroscience, systems and molecular neuroimaging, communication, language, and learning. In 2023, the average time from submission to initial decision for Neuroimage was approximately 9 weeks, and the average time from acceptance to online publication was around 0.5 weeks. Based on the sample of recently published articles, the majority of articles were reviewed and accepted within a review period of approximately 4–5 months.

**Table 1:** The top ten cited journals for functional magnetic resonance imaging research on depression

Ranking	Journal	Cocitation counts	JIF^a^ (2023)	Quartile in category ^a^ (2023)	JCI ^a^ (2023)
1	Neuroimage	3502	4.7	Q1	1.49
2	Biological Psychiatry	2889	9.6	Q1	2.25
3	Human Brain Mapping	2449	3.5	Q1	1.06
4	American Journal of Psychiatry	2330	15.1	Q1	3.76
5	Journal of Affective Disorders	2330	4.9	Q1	1.47
6	Proceedings of the National Academy of Sciences of the United States of America	2248	9.4	Q1	2.39
7	Journal of Neuroscience	2210	4.4	Q1	1.33
8	Plos One	2056	2.9	Q1	0.88
9	Neuropsychopharmacology	1829	6.6	Q1	1.6
10	Cerebral Cortex	1731	2.9	Q2	0.93

^a^JIF, Quartile in category and the JCI according to Journal Citation Reports (2023)

*JIF* journal impact factor, *Q* quartile, *JCI* journal citation indicator

### Analysis of cited references

In bibliometrics, the intellectual base of one discipline could be reflected by the references in these articles [27]. The top five most frequently cited references were shown in the Table 2 and Supplementary Fig. 2, indicating that these studies form the basis of fMRI research on depression and were of great interest to many researchers. The most cited reference was a meta-analysis of resting state functional connectivity in major depressive disorder [28]. This study discovered that individuals with depression have reduced connectivity within frontoparietal control systems. Additionally, there was imbalanced connectivity between control systems and networks associated with internal or external attention. These findings suggested that individuals with depression tend to focus more on internal thoughts and neglect engagement with the external world. The second most cited reference mentioned technological updates in data processing and analysis for resting state brain imaging, mentioning that the development of the toolkit DPABI makes data analysis less manual, less time, less skill required, less risk of accidental error, and more comparable between studies [29].

**Table 2:** Top five cocited references related to functional magnetic resonance imaging of depression research

Ranking	Representative author (publication year)	Cited reference	Cocitation counts
1	Kaiser RH (2015) [28]	Large-Scale Network Dysfunction in Major Depressive Disorder A Meta-analysis of Resting-State Functional Connectivity	131
2	Yan CG (2016) [29]	DPABI: Data Processing & Analysis for (Resting-State) Brain Imaging	105
3	Yan CG (2019) [30]	Reduced default mode network functional connectivity in patients with recurrent major depressive disorder	101
4	Drysdale AT (2017) [31]	Resting-state connectivity biomarkers define neurophysiological subtypes of depression	94
5	Sheline YI (2010) [32]	Resting-state functional MRI in depression unmasks increased connectivity between networks via the dorsal nexus	94

### Analysis of country

CiteSpace was used to perform a co-citation analysis of the countries (regions) (Supplementary Fig. 3). The distribution of publications across countries was highly uneven, with a clear "top-heavy" effect, as the majority of papers are authored by scholars from a few countries. Among them, scholars from the United States have contributed the most research papers in this field (with a total of 1477 publications), accounting for 36.48% of the total number of publications, and have received a high citation count of 62,949 (Supplementary Table 2). China ranks second, with a total of 1326 publications and 28,534 citations. Canada has the highest average citation count per paper, with 248 papers receiving 12,692 citations, with an average citation count of 51.18. Inter-country collaboration is quite frequent, resulting in a network of cooperation among developed countries centered around the United States, including Germany, the England, and Canada, as well as a network led by China, which includes Australia.

### Analysis of institution

**Fig. 3:**
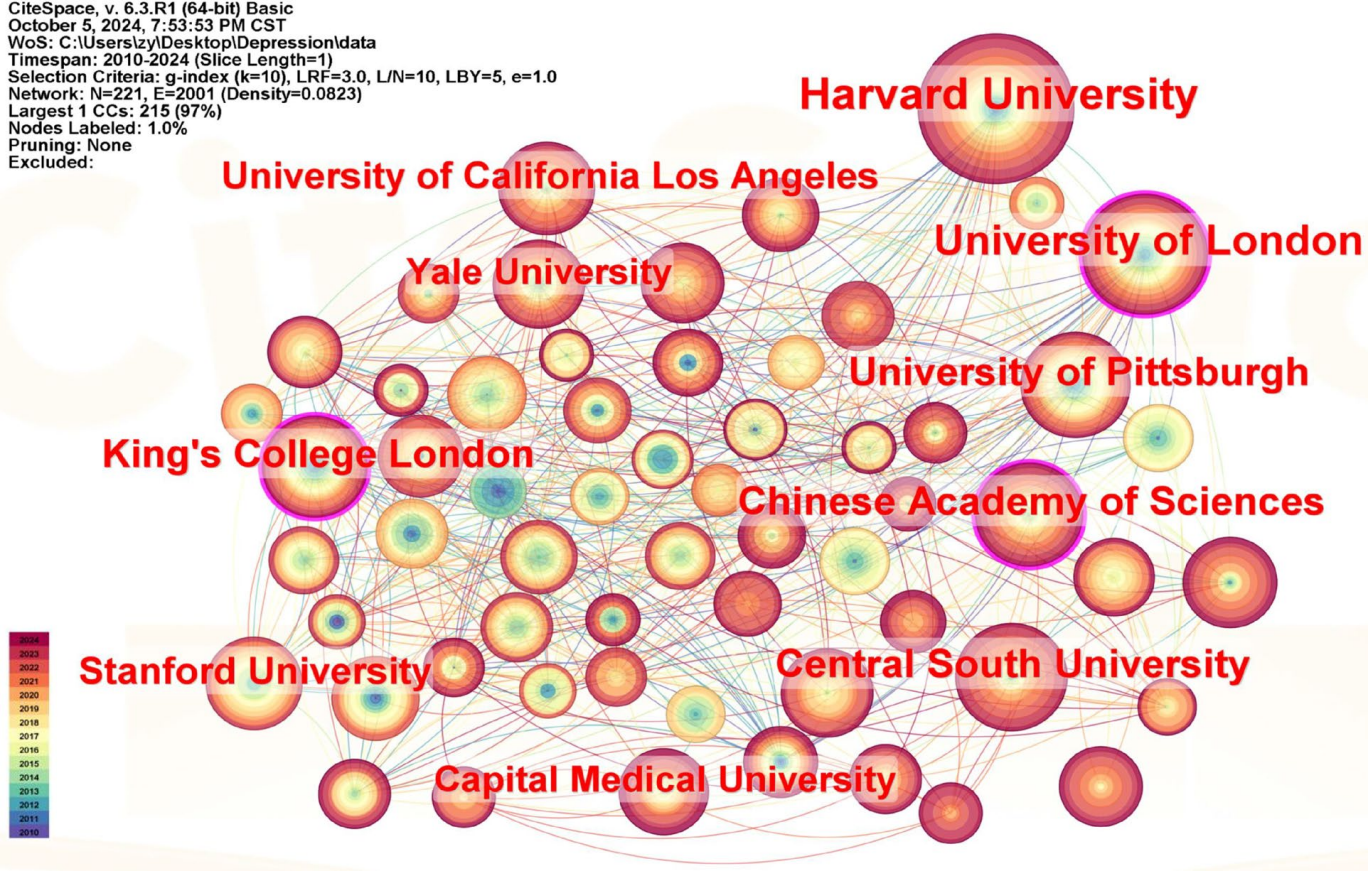
Map of institutions researching functional magnetic resonance imaging of depression from 2010 to 2024. The color represents the year, the size of the circle represents the number of publications, and the line represents the collaboration between the institutions

Cite Space was used to perform a co-citation analysis of the institutions (Fig. 3). The top 5 institutions with the highest number of publications were Harvard University (217), University of London (163), University of Pittsburgh (110), Chinese Academy of Sciences (99), and Central South University (97) (Supplementary Table 2). The Harvard University mainly studies transcranial magnetic stimulation therapy, neuroimaging grading of depression, and clinical application of neuroimaging in depression treatment. Among them, the most cited is the study by Michael D Fox et al., which found that antidepressant efficacy of different left dorsolateral prefrontal cortex transcranial magnetic stimulation sites is associated with the anticorrelation of each site with the subgenual cingulate [33].

### Analysis of keyword cocurrence and burst keywords

Keywords co-occurring knowledge maps could reflect hot topics over time, while burst keywords that are frequently quoted over a period of time may indicate frontier topics. A co-occurrence network of keywords was generated for 4,049 documents, and 26 key keywords with a frequency greater than 57 were selected for visualization. The top six keywords with the highest co-occurrence frequency were functional connectivity, meta analysis, prefrontal cortex, default mode network, amygdala, anterior cingulate cortex. The most popular keywords in recent studies included default mode network, prefrontal cortex, amygdala, anterior cingulate cortex, etc., indicating that the DMN was currently recognized as the brain network most closely related to depression in fMRI analysis, and that the prefrontal cortex, amygdala, anterior cingulate cortex, and other brain regions are closely related to depression (Supplementary Fig. 4).

Clustering analysis of keywords using log-likelihood ratio can clearly show the internal characteristics of the research object and provide reliable evidence for predicting the evolution of research hotspots. Keywords with a larger LLR are more representative of this cluster. We set the positive likelihood ratio ≥ 19, P ≤ 10^–4^ and top 15 as the screening conditions to screen the keywords. keywords were divided into four categories, as shown in Table 3. The hotspots in fMRI field on depression mainly focus on major depression, with a particular emphasis on the DMN. In addition, the functional connectivity of brain regions and the regional homogeneity of brain regions in the resting state of depressed patients [34] have also been research hotspots in recent years.

**Table 3:** Cluster analysis of keywords

Cluster ID	Keywords (Positive Likelihood Ratio, P)	Content
1	Functional magnetic resonance imaging (34.77, 1.0^–4^); major depressive disorder (33.44, 1.0^–4^); meta analysis (25.47, 1.0^–4^); magnetic resonance imaging (22.82, 1.0^–4^); major depression (22.79, 1.0^–4^) functional magnetic resonance imaging (101.4, 10^–4^); magnetic resonance imaging (97, 10^–4^); bipolar disorder (49.5, 10^–4^); regional homogeneity (26.23, 10^–4^); fMRI (23.63, 10^–4^)	Functional magnetic resonance imaging of major depression
2	Functional connectivity (127.56, 10^–4^); default mode network (91.22, 10^–4^); resting-state functional magnetic resonance imaging (31.64, 10^–4^); scale (21.96, 10^–4^); disorder (19.74, 10^–4^)	Default Mode Network
3	Major depressive disorder (47.54, 10^–4^); prefrontal cortex (45.15, 10^–4^); major depression (42.65, 10^–4^); functional connectivity (28.99, 10^–4^); cortex (22.72, 10^–4^)	Functional Connectivity
4	Activation (51.88, 10^–4^); fmri (45.52, 10^–4^); brain (39.64, 10^–4^); amygdala (33.61, 10^–4^); regional homogeneity (30.49, 10^–4^)	Regional homogeneity of the brain regions

**Fig. 4:**
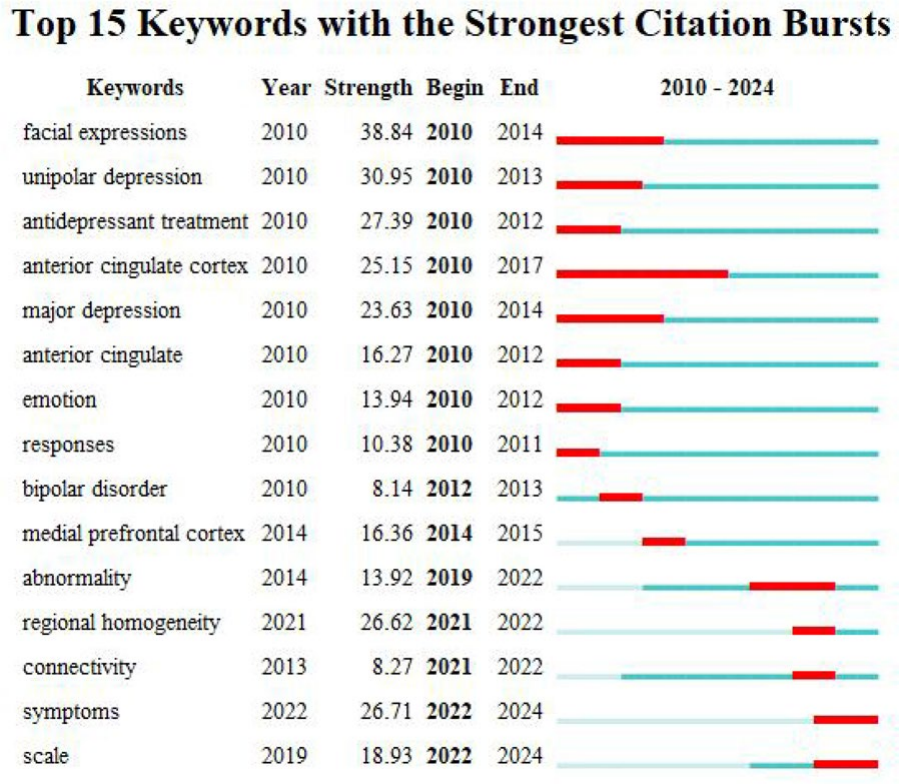
Top 15 keywords with the strongest citation bursts over time. One column represents one year. Red columns indicate that keywords are often cited, and the red column indicates the year in which the keyword outbreak was cited

In order to gain a clearer understanding of the sudden research hotspots in the fMRI field of depression, further analysis using Cite Space's Bursts function was conducted, with results shown in Fig. 4. From 2022 to 2024, studies in this field have tended towards symptions and scale, indicating the combination of clinical scale assessment and fMRI analysis of depressive symptoms is a new trend. The term with the highest burst intensity among the burst words is "facial expression," which is due to the prominent feature of depression being low mood, and facial expressions can provide a more intuitive way to perceive a person's emotions [35]. Some studies suggested that there were significant differences in facial behavior between individuals with depression and those without depression [36]. Therefore, facial expressions plays an important role in the treatment of depression. Studies have found that the perception of facial expressions is related to the fusiform gyrus and superior temporal sulcus. The amygdala, anterior insula, orbitofrontal cortex, and ventral striatum have been shown to be involved in the recognition of emotions and the generation of emotional responses in response to provocative stimuli [37]. From 2010 to 2017, the anterior cingulate/anterior cingulate cortex became a key area of research in depression. Some studies have found that the anterior cingulate cortex is a key central region for depression induced by pain [38]. Retrieval of autobiographical memories of painful events can activate the anterior cingulate cortex [39]. Suppressed activity of the rostral anterior cingulate cortex as a biomarker for depression remission [40]. The dorsal anterior cingulate cortex was associated with conflict errors and monitoring [41], including interference between top-down and bottom-up processes involved in emotion reappraisal and generation [42]. The anterior cingulate cortex and prefrontal cortical regions have been shown to play important roles in emotion regulation [43]. The medial prefrontal cortex became a research focus during the period of 2014–2015, and was believed to be involved in self-monitoring processes, including the evaluation of internal states relative to external stimuli [44].

### Meta-analysis

**Fig. 5:**
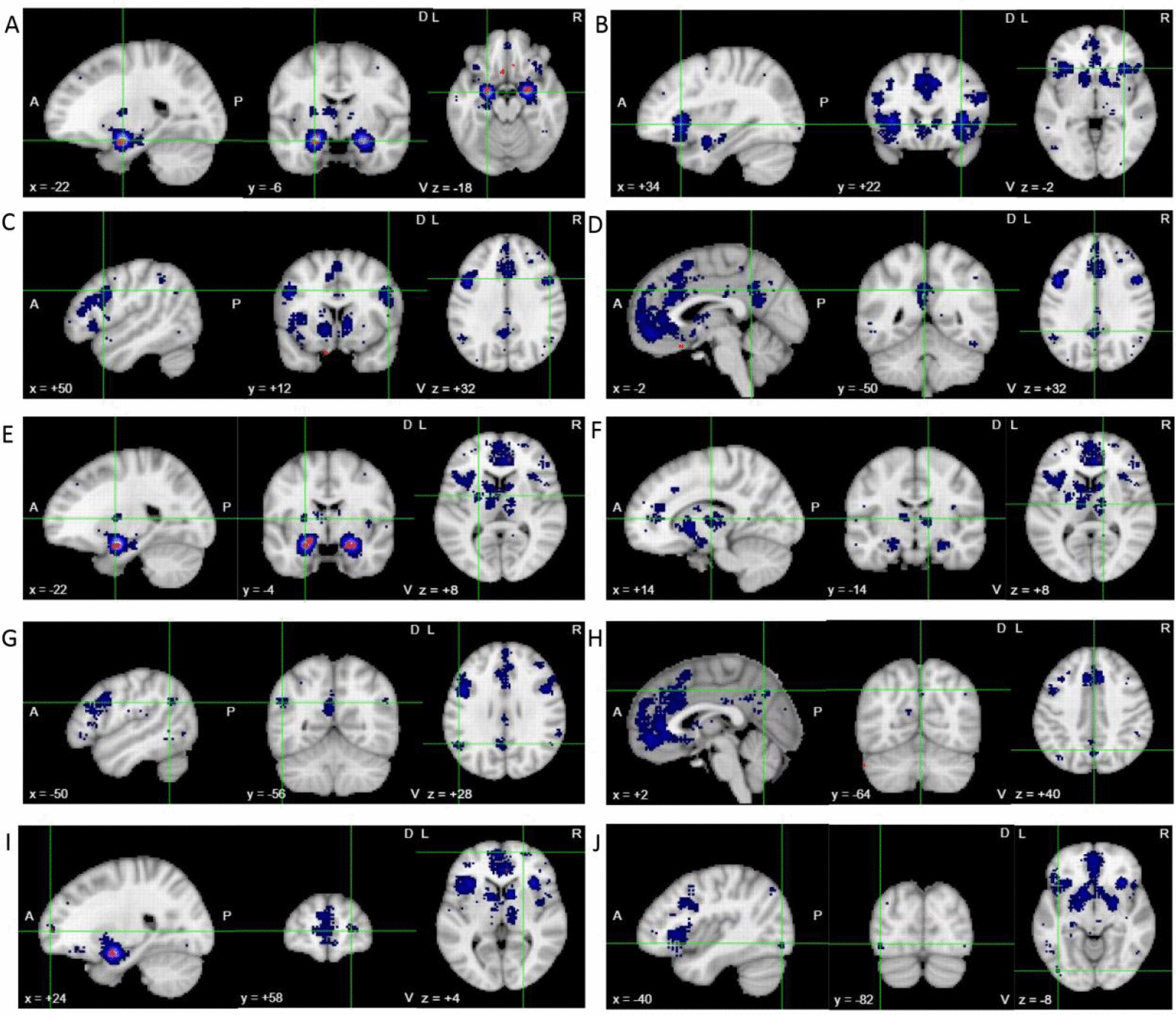
The top ten differences in brain regions between depression patients and healthy subjects (**A**. Amygdala_L; **B**. Insula_R; **C**. Pars opercularis gyri frontalis inferioris_R (Frontal_Inf_Oper_R); **D**. Posterior cingulate cortex_L (Cingulum_Post_L); **E**. Putamen_L; **F**. Thalamus_R; **G**. Angular gyrus_L (Angular_L); **H**. Precuneus_R; **I**. Dorsolateral superior frontal gyrus_R (Frontal_Sup_R); **J**. Inferior occipital gyrus_L (Occipital_Inf_L))

Meta-analysis was conducted on 502 studies with "depression" as the main subject heading. The brain region coordinates were extracted from the literature, and the meta-analysis was conducted to determine the different brain regions between depressed patients and healthy controls. The top ten brain regions identified were: Amygdala_L, Insula_R, Pars opercularis gyri frontalis inferioris_R, Posterior cingulate cortex_L, Putamen_L, Thalamus_R, Angular gyrus_L, Precuneus_R, Dorsolateral superior frontal gyrus_R, Inferior occipital gyrus_L (Fig. 5). The MNI peak coordinates, T values, and cluster sizes are presented in the Supplementary Table 3.

## Discussion

In this study, CiteSpace software was used to conduct a bibliometric analysis of the relevant citations in the fMRI of depression. The software was able to visualize the current status and trends in the field, providing a systematic understanding of the potential collaborations, hotspots, and frontiers. Additionally, a meta-analysis was conducted to identify the top ten brain regions where differences were observed between depressed patients and healthy controls. Between 2010 and 2024, there were a total of 4,049 studies on fMRI in depression. Especially after 2018, fMRI related to depression has been widely reported. During this period, the most cited article was "Resting-state connectivity biomarkers define neurophysiological subtypes of depression" [31]. In this study, the authors used fMRI in a large multi-site sample (n = 1188) to subdivide depressed patients into four neurophysiological subtypes ("biotypes") defined by different patterns of dysfunctional connectivity in the default mode and fronto-striatal networks. These biotypes could not be distinguished based solely on clinical features, but were associated with different clinical symptom features. This article has been cited 1286 times and was one of the most frequently cited papers in the relevant literature on fMRI in depression.

According to Price's law, the core author group in the field of fMRI research on depression has not yet formed. But there are still some high-productivity authors, such as Gong Qiyong, who often collaborate with Yuan Yonggui. They collaborated to study altered resting-state dynamic functional brain networks in major depressive disorder. At the same time, the uneven development of countries and institutions was also vividly reflected in this field. Leading countries in this field are predominantly developed nations, with American scholars contributing the most research papers, making them a major force in this area. Although China has also achieved significant results, other developing countries have yet to establish a substantial presence in research. This study also revealed numerous publications on units such as Harvard University, as well as their research interests, including transcranial magnetic stimulation therapy, neuroimaging grading of depression, and clinical application of neuroimaging in depression treatment, etc.. Some highly cited literature, high publication volume and high cited journals were also reported and discussed in detail in this study, and their impact was explained. After 2022, symptions and scale were emerging as new research trends in this field. This series of results provides a useful reference for studying fMRI of depression.

Hot topics in fMRI research on depression mainly focus on major depression, the DMN, functional connectivity and brain regions. The DMN includes the posterior cingulate cortex/precuneus, medial prefrontal cortex, and bilateral angular gyrus [45]. The DMN is a network of brain regions that is active when we are not engaged in any specific task [46]. DMN is involved in a diverse array of functions, such as episodic memory, monitoring the external environment, self-relevant mental processing, planning the future and remembering the past [47]. Indeed, some studies have explored the intrinsic relationships between the three core networks in depression and found that there was a decrease in functional connectivity between the DMN and the central executive network, while there was an increase in functional connectivity between the DMN and the salience network [48].

In 2022–2024, symptoms and scales became the hot keywords in the field, indicating that the combination of clinical scale evaluation and functional magnetic resonance analysis is a new trend in depressive symptoms. For instance, the combination of fMRI methods with clinical scale assessments can differentiate between subclinical depression and major depressive disorder patients [49]. Additionally, utilizing fMRI to calculate regional homogeneity allows for the evaluation of the relationship between changes in brain regional homogeneity and depressive symptoms in COVID-19 patients [50]. Furthermore, integrating scales with fMRI reveals the neurofunctional plasticity of medications for depression and potential biomarkers. These findings underscore the emerging focus on the cross-validation of clinical scale assessments and fMRI [51].

Functional connectivity of brain regions was one of the current research hotspots. With the development of neuroimaging meta-analysis, it has become possible to uncover the brain regions associated with depression. Our meta-analysis revealed that the top ten brain regions where depressed patients showed significant differences compared to healthy controls were the Amygdala_L, Insula_R, Frontal_Inf_Oper_R, Cingulum_Post_L, Putamen_L, Thalamus_R, Angular_L, Precuneus_R, Frontal_Sup_R, Occipital_Inf_L. Notably, amygdala, putamen, and thalamus are part of the reward circuit. The amygdala is a small, almond-shaped structure located deep within the temporal lobe of the brain [52]. It plays a critical role in processing emotions, particularly fear and anxiety [53]. Previous studies have shown that the amygdala is hyperactive in individuals with depression, which may contribute to the negative emotional bias and heightened sensitivity to stress that are characteristic of this disorder [54, 55]. The lenticular putamen is a subcortical structure located in the basal ganglia, which is involved in the regulation of movement and reward processing [56]. Previous studies has shown that abnormalities in the functioning of the basal ganglia, including the lenticular putamen, may contribute to the anhedonia (loss of pleasure) and other symptoms of depression [56]. The thalamus is an important subcortical structure that receives sensory information and relays it to the appropriate parts of the cerebral cortex [57]. Previous studies have suggested that the thalamus may be involved in the regulation of mood and emotion, and abnormalities in thalamic functioning have been observed in individuals with depression [58, 59].

The posterior cingulate cortex, precuneus, and angular gyrus are part of the DMN. The posterior cingulate is involved in myriad cognitive functions, including episodic memory retrieval and spatial memory [60]. Abnormalities in the activity and connectivity of the posterior cingulate gyrus have been observed in individuals with depression, suggesting that this region may play a role in the pathophysiology of the disorder [61]. The precuneus is the superior parietal part of the medial surface of each cerebral hemisphere, and is located in front of the cuneus (the superior part of the occipital lobe) [62]. The precuneus is involved in a wide range of cognitive processes, including visual-spatial imagery, episodic and autonoetic memory, as well as self-related processing [63]. The angular gyrus is involved in a range of functions, including language processing, spatial cognition, and the integration of sensory information [64]. Some studies have suggested that the angular gyrus may be involved in the pathophysiology of depression, particularly in relation to the modulation of emotional responses and the regulation of cognitive processes [43, 65].

The insula belongs to the salience network. The insula is a structure that connects temporal and frontal lobes deep within the lateral (Sylvian) sulcus. It is highly active in many different task situations, including cognition, interoception, perception, and emotion [66]. There is evidence to suggest that the insula may play a role in the pathophysiology of depression, particularly in relation to the regulation of emotional responses and the integration of sensory and emotional information [67].

The pars opercularis gyri frontalis inferioris belongs to the dorsal attention network. It is a component of the inferior frontal gyrus, which represents the pre-language system involved in language comprehension and production, but is also related to general cognitive and perceptual functions, including attention, working memory, inhibition control, planning/goal-directed behavior, fluid intelligence, and consciousness [68]. Research suggests that the inferior frontal gyrus cortex contributes to emotional processing in the human brain [69].

The dorsolateral superior frontal gyrus belongs to the frontoparietal network. It has long been considered a core area for higher cognitive functions, including attention, working memory, and cognitive control [70]. Previous studies have suggested that abnormalities in dorsolateral prefrontal functioning may contribute to the cognitive deficits observed in individuals with depression [71]. The inferior occipital gyrus belongs to the visual area. The inferior occipital gyrus is located in the occipital lobe, which is primarily involved in visual processing [72].

While all of these brain regions are involved in different functions, they are interconnected and work together to regulate various aspects of cognition, emotion, and behavior. Further research is needed to fully understand the complex relationship between brain function, depression, and the various regions involved in these processes. Previous studies have also indicated that depression was a manifestation of disrupted brain networks [73]. Many fMRI studies have reported abnormal functional connectivity in multiple resting-state networks of patients with unipolar or bipolar depression compared to control groups. These abnormalities in functional connectivity were characterized by either increased or decreased connectivity between brain regions, indicating a state of network disarray [73].

## Limitation

CiteSpace primarily analyzes data directly obtained from Web of Science, rather than literature selected by reference management software. Therefore, it requires the data included in bibliometric analysis to be as accurate and highly matched as possible, rather than being systematic and comprehensive. In order to meet this requirement, we conducted pre-retrievals using different search methods, such as subject term, title, and full text, to compare the data obtained from different search methods with the match of our target study. Ultimately, we chose the subject term retrieval method. This may result in some related studies being omitted. Additionally, because CiteSpace cannot exclude the phenomenon of "citation cartels" among authors, an analysis focused solely on publication volume and collaboration networks may hinder the identification of other potentially high-output authors, thereby obstructing the clinical translation of the data.

## Conclusion

This study employed bibliometric analysis to systematically analyze and visualize the fundamental characteristics of fMRI studies related to depression. A meta-analysis of current fMRI results reveals differences in brain regions between depressed patients and healthy controls. These findings may assist medical researchers in identifying new directions and reestablishing collaborations.

## Supplementary Information

Additional file 1: Table 1. Top 10 active authors in functional magnetic resonance imaging of depression according to the publications. Table 2. Top 5 prolific countries or institutions researching functional magnetic resonance imaging of depression. Table 3. Differences in brain region information between depression patients and healthy subjects ranked in the top ten. Figure 1. Journal cocitation map related to functional magnetic resonance imaging of depression from 2010 to 2024. Figure 2. The highly cited references reflect the knowledge base of fMRI for depression from 2010 to 2024. Figure 3. Map of countries researching functional magnetic resonance imaging of depression from 2010 to 2024. The color represents the year, the size of the circle represents the number of publications, and the line represents the collaboration between the countries. Figure 4. A keyword cooccurrence map of functional magnetic resonance imaging of depression from 2010 to 2024

## Data Availability

The datasets or software generated and/or analysed during the study are available in the Web of Science: http://apps.webofknowledge.com/; Neurosynth: http://www.neurosynth.org; CiteSpace software: http://cluster.ischool.drexel.edu/ ~ cchen/citespace/.
