# Supplementary material for: Functional magnetic resonance imaging of depression: a bibliometrics and meta-analysis
Source: Ann Gen Psychiatry. 2024 Oct 24;23:39. doi: 10.1186/s12991-024-00525-x (PMC11520125; doi:10.1186/s12991-024-00525-x)
Supplement: Supplementary file 1 — Additional file 1: Table 1. Top 10 active authors in functional magnetic resonance imaging of depression according to the publications. Table 2. Top 5 prolific countries or institutions researching functional magnetic resonance imaging of depression. Table 3. Differences in brain region information between depression patients and healthy subjects ranked in the top ten. Figure 1. Journal cocitation map related to functional magnetic resonance imaging of depression from 2010 to 2024. Figure 2. The highly cited references reflect the knowledge base of fMRI for depression from 2010 to 2024. Figure 3. Map of countries researching functional magnetic resonance imaging of depression from 2010 to 2024. The color represents the year, the size of the circle represents the number of publications, and the line represents the collaboration between the countries. Figure 4. A keyword cooccurrence map of functional magnetic resonance imaging of depression from 2010 to 2024 [file 12991_2024_525_MOESM1_ESM.docx]

**Supplementary Table 1** Top 10 active authors in functional magnetic resonance imaging of depression according to the publications

| Rank | author | documents | citations | Average Citation/Publication | Centrality |
| --- | --- | --- | --- | --- | --- |
| 1 | Gong, Qiyong | 24 | 2655 | 110.63 | 0 |
| 2 | Yuan, Yonggui | 18 | 868 | 48.22 | 0 |
| 3 | Phillips, Mary L. | 21 | 2281 | 108.62 | 0 |
| 4 | Shao, Yi | 34 | 270 | 7.94 | 0 |
| 5 | Pizzagalli, Diego A. | 20 | 1407 | 70.35 | 0 |
| 6 | Veltman, Dick J. | 18 | 2172 | 120.67 | 0.01 |
| 7 | Chen, Huafu | 15 | 1485 | 99.00 | 0 |
| 8 | Guo, Wenbin | 13 | 1284 | 98.77 | 0.01 |
| 9 | Harmer, Catherine J. | 6 | 1808 | 301.33 | 0 |
| 10 | Liu, Feng | 13 | 940 | 72.31 | 0 |

**Supplementary Table 2** Top 5 prolific countries or institutions researching functional magnetic resonance imaging of depression

| Rank | Country/Institution | Documents | Centrality | Citations | Average Citation/Publication |
| --- | --- | --- | --- | --- | --- |
| 1 | USA | 1477 | 0.33 | 62949 | 42.62 |
| 2 | Peoples R China | 1326 | 0.35 | 28534 | 21.52 |
| 3 | Germany | 488 | 0.17 | 23256 | 47.66 |
| 4 | England | 383 | 0.10 | 18036 | 47.09 |
| 5 | Canada | 248 | 0.16 | 12692 | 51.18 |
| 6 | Harvard University | 217 | 0.1 | 11793 | 54.35 |
| 7 | University of London | 163 | 0.16 | 9012 | 55.29 |
| 8 | University of Pittsburgh | 110 | 0.03 | 5779 | 52.54 |
| 9 | Chinese Academy of Sciences | 99 | 0.06 | 3796 | 38.34 |
| 10 | Central South University | 97 | 0.01 | 3544 | 36.54 |

**Supplementary Table 3.** Differences in brain region information between depression patients and healthy subjects ranked in the top ten.

| Area | MNI peak coordinates | | | T value | Cluster Size |
| --- | --- | --- | --- | --- | --- |
| Amygdala_L | -22 | -6 | -18 | 24.05 | 7616 |
| Insula_R | 34 | 22 | -2 | 12.72 | 698 |
| Frontal_Inf_Oper_R | 50 | 12 | 32 | 8.64 | 652 |
| Cingulum_Post_L | -2 | -50 | 32 | 8.19 | 313 |
| Putamen_L | -22 | -4 | 8 | 6.83 | 48 |
| Thalamus_R | 14 | -14 | 8 | 6.38 | 155 |
| Angular_L | -50 | -56 | 28 | 5.93 | 50 |
| Precuneus_R | 2 | -64 | 40 | 5.93 | 29 |
| Frontal_Sup_R | 24 | 58 | 4 | 5.47 | 25 |
| Occipital_Inf_L | -40 | -82 | -8 | 5.02 | 14 |


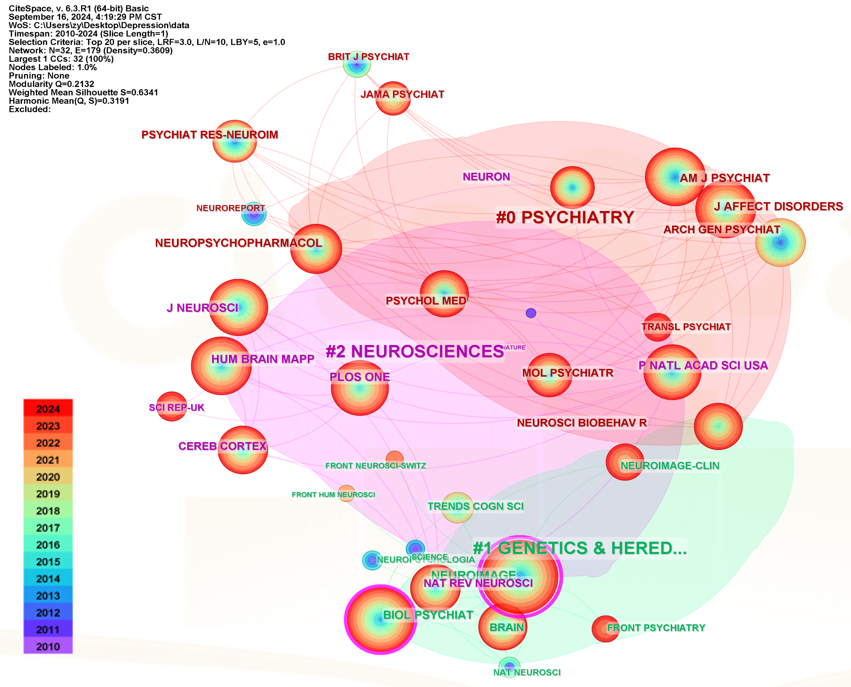


**Supplementary Figure 1.** Journal cocitation map related to functional magnetic resonance imaging of depression from 2010 to 2024.


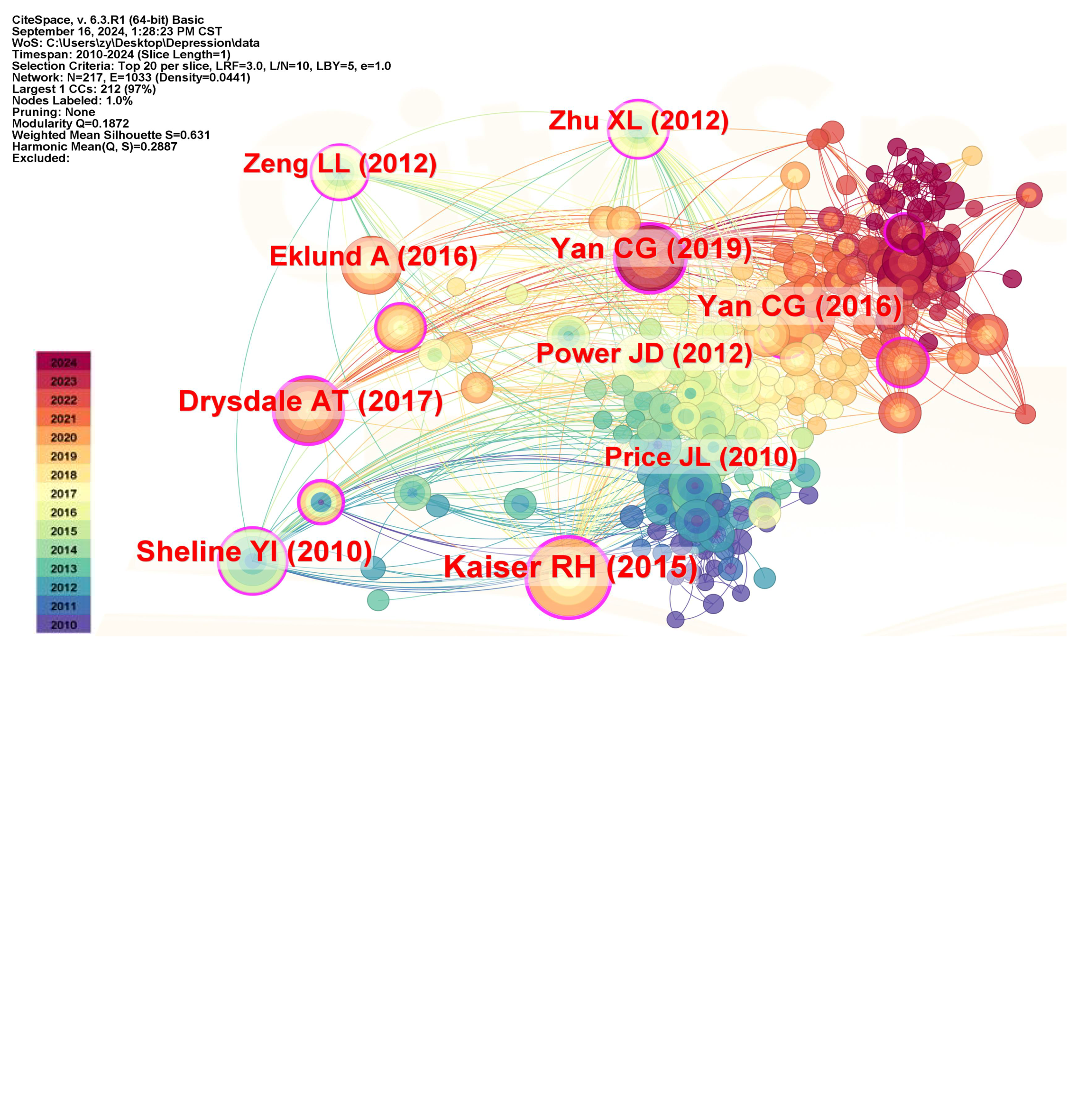


**Supplementary Figure 2.** The highly cited references reflect the knowledge base of fMRI for depression from 2010 to 2024.


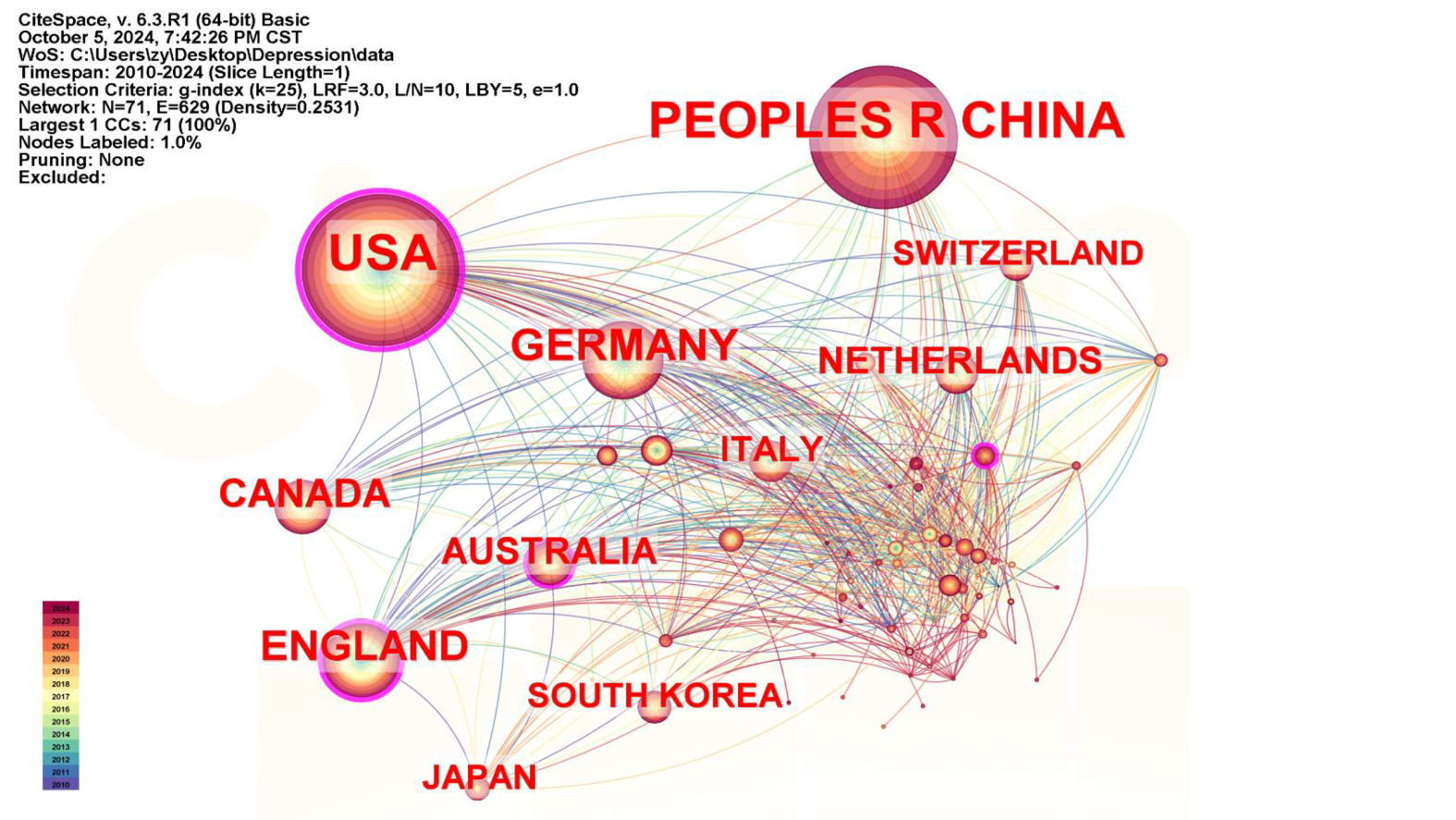


**Supplementary Figure 3.** Map of countries researching functional magnetic resonance imaging of depression from 2010 to 2024. The color represents the year, the size of the circle represents the number of publications, and the line represents the collaboration between the countries.


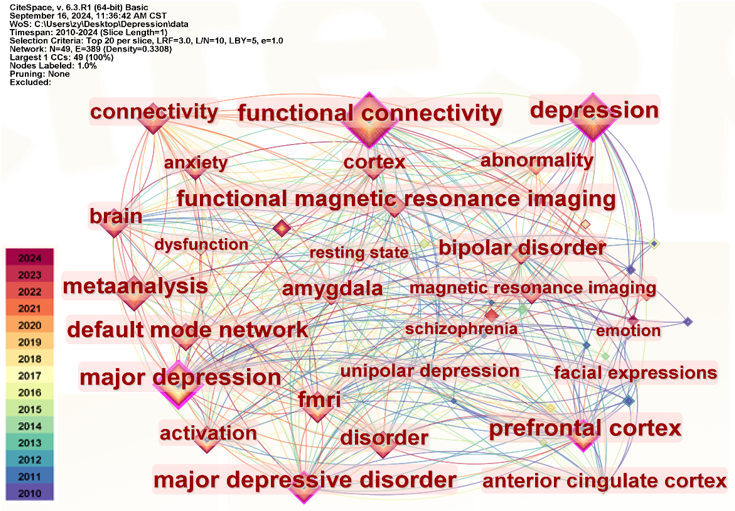


**Supplementary Figure 4**. A keyword cooccurrence map of functional magnetic resonance imaging of depression from 2010 to 2024.
